# Low-Frequency Repetitive Transcranial Magnetic Stimulation Restores Dynamic Functional Connectivity in Subcortical Stroke

**DOI:** 10.3389/fneur.2021.771034

**Published:** 2021-12-07

**Authors:** Yin Qin, Xiaoying Liu, Xiaoping Guo, Minhua Liu, Hui Li, Shangwen Xu

**Affiliations:** ^1^Fuzong Clinical Medical College of Fujian Medical University, Fuzhou, Fujian, China; ^2^Department of Rehabilitation Medicine, The 900th Hospital of Joint Logistic Support Force, PLA, Fuzhou, China; ^3^Department of Radiology, The 900th Hospital of Joint Logistic Support Force, PLA, Fuzhou, China

**Keywords:** subcortical stroke, transcranial magnetic stimulation, dynamic functional connectivity, resting-state functional magnetic resonance imaging, time-varying connectivity

## Abstract

**Background and Purpose:** Strokes consistently result in brain network dysfunction. Previous studies have focused on the resting-state characteristics over the study period, while dynamic recombination remains largely unknown. Thus, we explored differences in dynamics between brain networks in patients who experienced subcortical stroke and the effects of low-frequency repetitive transcranial magnetic stimulation (LF-rTMS) on dynamic functional connectivity (dFC).

**Methods:** A total of 41 patients with subcortical stroke were randomly divided into the LF-rTMS (*n* = 23) and the sham stimulation groups (*n* = 18). Resting-state functional MRI data were collected before (1 month after stroke) and after (3 months after stroke) treatment; a total of 20 age- and sex-matched healthy controls were also included. An independent component analysis, sliding window approach, and k-means clustering were used to identify different functional networks, estimate dFC matrices, and analyze dFC states before treatment. We further assessed the effect of LF-rTMS on dFCs in patients with subcortical stroke.

**Results:** Compared to healthy controls, patients with stroke spent significantly more time in state I [*p* = 0.043, effect size (ES) = 0.64] and exhibited shortened stay in state II (*p* = 0.015, ES = 0.78); the dwell time gradually returned to normal after LF-rTMS treatment (*p* = 0.015, ES = 0.55). Changes in dwell time before and after LF-rTMS treatment were positively correlated with changes in the Fugl–Meyer Assessment for Upper Extremity (pr = 0.48, *p* = 0.028). Moreover, patients with stroke had decreased dFCs between the sensorimotor and cognitive control domains, yet connectivity within the cognitive control network increased. These abnormalities were partially improved after LF-rTMS treatment.

**Conclusion:** Abnormal changes were noted in temporal and spatial characteristics of sensorimotor domains and cognitive control domains of patients who experience subcortical stroke; LF-rTMS can promote the partial recovery of dFC. These findings offer new insight into the dynamic neural mechanisms underlying effect of functional recombination and rTMS in subcortical stroke.

**Registration:** http://www.chictr.org.cn/index.aspx, Unique.identifier: ChiCTR1800019452.

## Introduction

Extensive overall structural and functional changes are observed after a stroke (1). Resting-state functional MRI (rs-fMRI) has strongly contributed to an understanding of the brain mechanism after a subcortical stroke (2). Studies on rs-fMRI have shown that functional connectivity (FC) and network indicators are disrupted during stroke (3). Moreover, low-frequency repetitive transcranial magnetic stimulation (LF-rTMS) combined with rehabilitation training can promote motor function recovery and cognitive enhancement and change the activation and FC of these networks (4, 5). However, the methods used in previous studies ignored temporal changes in the fMRI signals; it is unclear whether FCs between brain networks change over time and how these changes are related to the LF-rTMS mechanism underlying the regulation of functional recovery in patients who have experienced a subcortical stroke.

With the emergence of dynamic FC (dFC) analyses, the time resolution of rs-fMRI data has been improved. dFC analyses are different from the previous resting-state connectivity analysis, which assumes that the FC of brain regions remains unchanged during the whole scanning process; dFC analysis makes full use of the dynamic information of the blood oxygen level-dependent (BOLD) signals in the time dimension to explain the variability of connections between brain regions over a short period of time (6, 7). An increasing number of studies have suggested that dFC may be related to behavior and can be used as a new biomarker of neurodegenerative diseases (8, 9). For example, Fiorenzato et al. (10) conducted a dynamic connection analysis in patients with Parkinson's disease and found that dementia in these patients stayed in a discrete state for the longest time, confirming that the emergence of a specific state of dynamic connection was significantly correlated with the severity of clinical symptoms. Similarly, Schumacher et al. (11) revealed a significant increase in the incidence of sparse connections in patients with Alzheimer's disease, which cannot be detected in static analysis.

As dFC analysis can extract more time-varying characteristics of information exchange between brain regions on a time scale and because these characteristics are significantly related to many physiological parameters (12), pathological features (13), and even intervention effects (14), dFC analysis seems to be particularly suitable for evaluating the complex and changeable characteristics of brain networks after stroke and exploring the neural mechanism of functional rehabilitation. Previous studies have shown that the proportion between integrated and segregated states was not balanced in patients who suffered from strokes (4) and that the temporal dynamics of FC were closely related to clinical severity (15, 16). However, these studies focus mainly on patients with acute or chronic stroke; there are few reports on the dynamic relationship between brain networks in patients with a convalescence period of 1–3 months after stroke. The initial 1–3 months after stroke remain the golden period of functional rehabilitation, during which effective rehabilitation measures and treatment strategies are particularly important to achieve maximum motor rehabilitation. LF-rTMS can regulate brain function reorganization and promote exercise recovery, but the mechanism of LF-rTMS is very complicated. Researchers have found that only considering task states and static FC are not enough to explain the time-varying dynamic information interaction of the brain and the dynamics of functional connections can better reveal its mechanism. Therefore, it is necessary to further study the dynamic changes in the characteristics of brain network interaction over time in patients with convalescent stroke and to explore whether LF-rTMS affects the temporal and spatial characteristics of dFC.

Therefore, we created a multinetwork model, including the sensorimotor network (SMN), visual network (VIS), auditory network (AUN), default mode network (DMN), frontoparietal network (FPN), and cerebellar (CB) network, and implemented the sliding window approach to compare the changes in dFC on rs-fMRI between patients with stroke and healthy controls (HCs). Moreover, we further explored whether LF-rTMS affects dFC in patients who suffered from a subcortical stroke. We hypothesized that the temporal and spatial characteristics of dFC in patients with convalescent stroke are different from that of HCs and that LF-rTMS can regulate these abnormalities and promote motor rehabilitation.

## Materials and Methods

### Participants

The experimental protocol was approved by the Medical Ethics Committee of the Fuzhou General Hospital (NO.2015011, ChiCTR1800019452) and written informed consent was obtained from all the participants. Inclusion criteria were: (a) first-onset ischemic stroke, (b) right-handedness before stroke, (c) a single lesion in the basal ganglia and adjacent regions, (d) a time interval between the first MRI examination and stroke onset of ~1 month before treatment, and (e) a time interval between the second MRI examination and stroke onset of ~3 months after treatment. Exclusion criteria were: (a) recurrent stroke, (b) any other brain abnormalities on MR images, (c) the modified Fazekas scale for white matter hyperintensities >1 (17), and (d) a history of any other neurological or psychiatric disorders. A total of 48 patients who experienced a subcortical stroke and agreed to participate in this study were included and were randomly assigned to either the LF-rTMS (24 patients) group or the sham rTMS group (24 patients). Seven patients were excluded for shedding during treatment (*n* = 7). Finally, 41 patients who experienced subcortical stroke were enrolled, comprising 23 patients (15 males; mean age: 58.52 ± 10.21 years) who underwent rTMS and 18 patients (10 males; mean age: 61.61 ± 7.66 years) who underwent sham stimulation. These patients were recruited from the Fuzhou General Hospital. Moreover, 20 HCs with matching age, sex, and hand habits were recruited, none of whom had a history of neurological or psychiatric illnesses.

### Study Design

**Figure 1:**
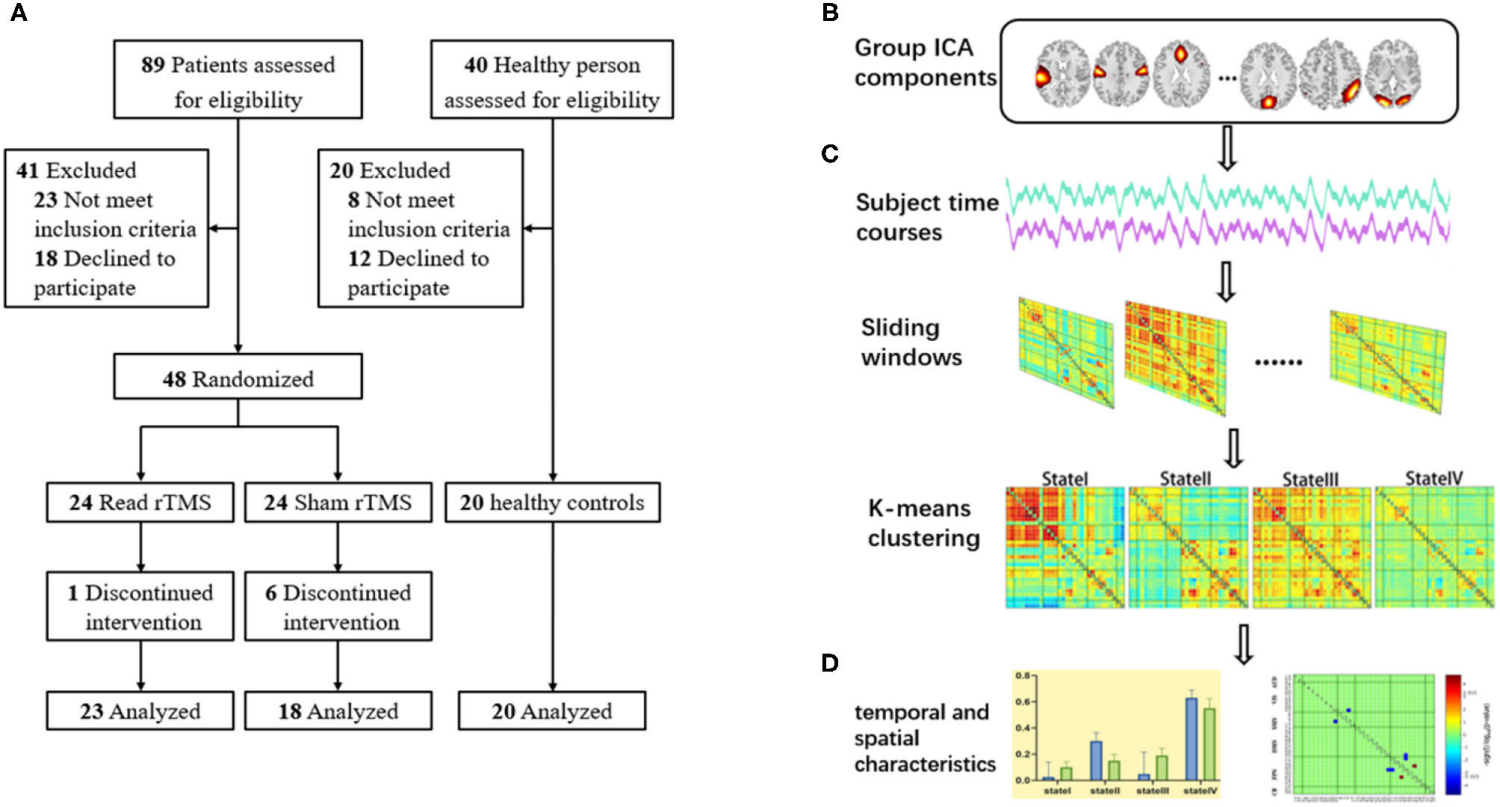
The schematic diagram of the research design. **(A)** Screening of participants; **(B)** Group independent component analysis; **(C)** sliding window approach and clustering analysis; **(D)** Calculate the temporal indicators and spatial characteristics of FC states.

Our study pipeline is shown in Figure 1. There are four major procedures in this framework: (A) Stroke subjects were randomized to either the LF-rTMS group and the sham rTMS group; 20 age- and sex-matched healthy controls were also included; (B) An independent component analysis to extract different functional networks; (C) Calculate dFNC between the independent components (IC) via a sliding window approach and perform a k-means clustering on dFNC estimates to identify reoccurring connectivity patterns (i.e., reoccurring states) across subjects and time; (D) Calculate the temporal indicators and spatial characteristics of FC states to explore differences in dynamics (Figure 1).

### Magnetic Resonance Imaging Acquisition

Magnetic resonance imaging examinations were performed using the 3.0 Tesla Siemens Tim Trio Scanner with a 12-channel head coil. All the subjects completed a 6-min resting-state MRI scan, during which they were required to stay awake, quiet, and close their eyes. The rs-fMRI data of each participant were acquired using the following gradient echo single echo-planar imaging sequences: repetition times (TR) = 2,000 ms, echo time = 21 ms, thickness = 4.0 mm, gap = 0.8 mm, field of view = 240 × 240 mm^2^, matrix = 64 × 64, and slices = 33. Three-dimensional T1-weighted images were obtained using the following sequences: repetition time = 1,900 ms, echo time = 2.52 ms, thickness = 1.0 mm, no gap, field of view = 240 × 240 mm^2^, matrix = 256 × 256, and slices = 176. Routine clinical sequences, such as T2-fluid-attenuated inversion recovery (T2-FLAIR) and diffusion-weighted imaging (DWI), were used to exclude other craniocerebral diseases.

### Rehabilitation Intervention

All the patients with stroke received routine rehabilitation training including a good limb position, passive joint movement, muscle stretch training, and neurodevelopmental therapy; each session was 40 min in length.

Low-frequency repetitive transcranial magnetic stimulation treatment: Before routine rehabilitation, the LF-rTMS group was stimulated with an air-cooled figure-8 coil of the transcranial magnetic stimulator (Magstim Corporation Ltd., Whitland, UK). The motor threshold (MT) of the primary motor area (M1) was measured before treatment, with patients relaxed and sitting quietly. The electrode was placed on the ventral side of the thenar muscle to stimulate the M1 area of the contralateral cerebral hemisphere. The minimum stimulus with no less than five evoked motor potentials > 50 μV in 10 consecutive stimuli was defined as the MT (18). During treatment, the patient was in the supine position and the stimulation frequency was 1 Hz, with an intensity of 90% MT. Each sequence had 10 pulses and the sequence interval was 2 s for a total of 1,200 pulses.

Sham stimulation: The sham stimulation group underwent rTMS with the same parameters (including position, stimulation frequency, interval time, and pulse number) as the LF-rTMS group. However, during intervention, the coil was inverted on the surface of the skull to ensure that it had the same stimulation sound without any effective stimulation.

The treatment period was once a day for 5 days a week over 8 weeks.

### Behavioral Assessments

For patients in the convalescence period of 1–3 months after stroke onset, the Fugl–Meyer Assessment for Upper Extremity (FMA-UE) was used to evaluate the motor ability of the upper limbs and the modified Barthel Index (MBI) was used to evaluate the self-care ability of patients in their daily life.

### Data Preprocessing

All the rs-fMRI data were preprocessed using the SPM12 software and the CONN toolbox (https://www.nitrc.org/projects/conn). Before image data preprocessing, we flipped the rs-fMRI data of the lesions in the left hemisphere from the left to the right along the median sagittal line to ensure that the lesions were uniformly located on the right side. Subsequently, the first five points were removed and slice timing, realignment, registration of the high-resolution three-dimensional (3D) anatomical image, and data normalization to the Montreal Neurological Institute (MNI) space were conducted. For resampling, the voxel size was 3 × 3 × 3 mm. Subsequently, the images were smoothed using a Gaussian kernel at half-maximum intensity and a full width of 6 × 6 × 6 mm to minimize the potential impact of head movement on dFC. According to the three displacements and three rotation parameters obtained in the head motion correction step for each subject, the average values of the displacement and rotation directions between two consecutive time point images were calculated. The exclusion criterion for this analysis were subjects whose average framework displacement was > 1.5 mm; however, in this study, no subjects were excluded.

### Outlining Stroke Lesions on DWI Data

**Figure 2:**
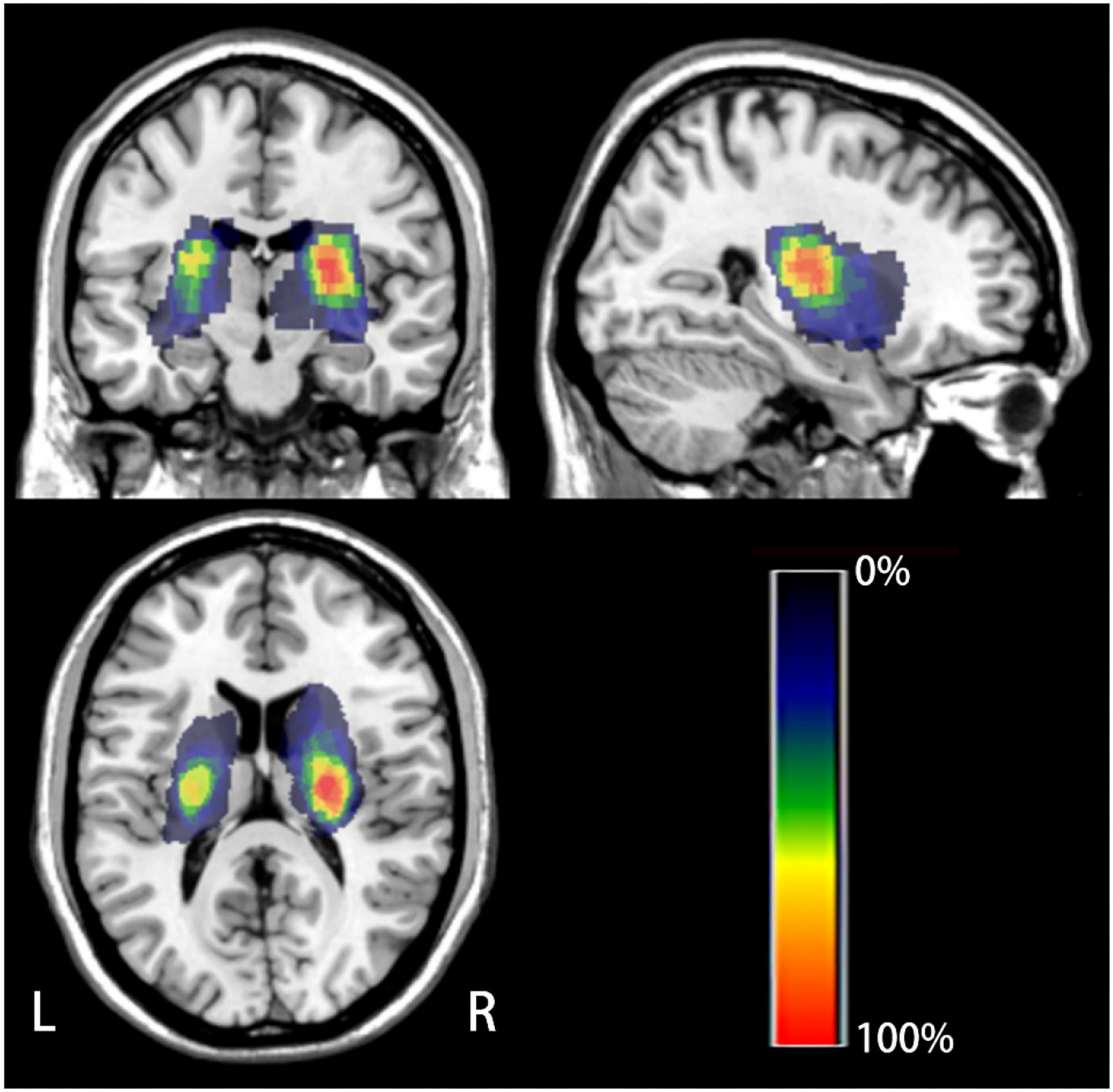
The lesion probability map of all the patients with stroke. The color indicates the frequency of superposition.

We used the SPM8 software) and the MRIcron software (http://www.mricro.com/mricron) to evaluate location of lesion. Two experienced radiologists used tools from the MRIcron software to manually track the lesion location in DWI images in patients who experienced acute infarction, taking the average of the data from two patients and accordingly estimating the region of interest (ROI). Subsequently, using MR segment-normalization in the SPM8 clinical toolbox, the ROI image was standardized to the MNI spatial template. Finally, the lesion map of patients with stroke was binarized and overlaid on the T1-weighted template in the MRIcron software to create a lesion probability map. We constructed a composite ROI including patients with stroke by using standardized ROIs and subsequently overlaid the composite ROI on a T1-weighted template to show the overlapping area (Figure 2).

### Group Independent Component Analysis (GICA)

The aforementioned preprocessed data were analyzed by GICA using the GIFT toolbox in MATLAB (http://mialab.mrn.org/software/gift/) for processing. The specific processing steps for spatial GICA were as follows: data dimensionality was reduced, the principal components were separated, and the infomax algorithm was used to evaluate 100 components of the data of each subject. In ICASSO, the independent component analysis algorithm was repeated 10 times to ensure the reliability and stability of the infomax independent component analysis algorithm (19). The spatial maps and time courses specific to each subject were obtained using back reconstruction. Subsequently, we conducted the Fisher's z-transformation; the data after the Fisher's z-transformation partly followed the normal distribution, wherein the mean was equal to the SD. Using the sorting GUI module Stanford functional ROIs of the GIFT software as the template (http://findlab.stanford.edu/functional_ROIs.html), the network components, which were remarkably similar to the template, were selected; these components were located on the gray matter and had low spatial overlap with the ventricles and blood vessels. Finally, 32 components were selected from 100 IC as intrinsic connectivity networks.

### Dynamic Functional Connectivity Analysis

We used the time dFC analysis module of the GIFT software toolbox for analysis, which mainly included the following steps: 32 network components were selected for analysis in the Setup/Run dFC module; postprocessing was subsequently performed on the previously preprocessed data, including detrending, despiking, and low-pass filtering at 0.15 Hz (20); and regression was conducted for head motion, age, and sex-related parameters. Previous studies have shown that window widths of 30–60 s can be successfully estimated for dFCs without noise control (21). We chose the general parameter setting with a window width of 22 TRs and step of 1 TR (22). Within each of these windows, we calculated dFCs from the L1 regularized exactness matrix. Finally, the FC matrixes were z-transformed to stabilize the variance for further analyses.

Recurring functional connection states were evaluated using k-means clustering on the window covariance matrix. During the analysis, the Euclidean distance, i.e., 500 iterates and 150 replicates, was used to estimate the resemblance between FC matrices (22). The optimal cluster number was estimated to be four (*k* = 4) using the elbow criterion of the clustering effectiveness index, which was calculated as the ratio of the intracluster distance to the intercluster distance.

### Demographic and Clinical Scales Analysis

Statistical analyses were conducted using the SPSS software version 20.0 (IBM Corporation, Armonk, New York, USA). Measurement data conforming to normal distribution are presented as mean ± SD. The one-way ANOVA was used for comparisons of three groups. The paired *t*-test was used for intragroup comparisons and the chi-squared test was used for sex comparisons. *p* < 0.05 was considered as statistically significant.

### Analysis of Temporal Indicators

We compared the following temporal indicators of the state vector of each participant: (a) fractional window for each state, which was defined as the proportion of all the windows in each state; (b) mean dwell time (MDT), which was defined as the mean number of subsequent windows under the same state; and (c) the number of transitions, which was defined as the total number of changes between states. The two-sample *t*-test was used to assess the differences between patients with stroke and HCs and the paired *t*-test was used for intragroup assessment (*p* < 0.05).

### Brain-Behavioral Correlation Analysis

We examined the potential association between dFC changes and motor function scores in patients with stroke. The Pearson correlation analysis was used to analyze the fractional window, MDT, number of transitions, and the behavioral FMA-UE scores of patients with stroke.

## Results

### Demographics and Clinical Characteristics

In total, 23 patients who experienced a subcortical stroke were treated with LF-rTMS (15 males; mean age, 58.52 ± 10.21 years), 18 patients who experienced a subcortical stroke were treated using sham rTMS (10 males; mean age, 61.61 ± 7.66 years), and 20 HCs (12 males; mean age, 59.90 ± 7.61 years) were included in this study. There was no significant difference in sex, age, and years of education between the three groups (Table 1). With respect to clinical data, there were no significant differences between the LF-rTMS group and the sham rTMS group at 1 month after stroke onset. However, the LF-rTMS group showed statistically significant improvements in the FMA-UE scores (*p* = 0.041) and MBI scores (*p* = 0.001) compared to the sham rTMS group at posttreatment (Table 2).

**Table 1:** Demographic and clinical information of patients with stroke and healthy controls.

**Variables**	**LF-rTMS**	**Sham rTMS**	**Healthy control**
	**(*n* = 23)**	**(*n* = 18)**	**(*n* = 20)**
Age, y	58.52 ± 10.21 (38–74)	62.27 ± 8.28 (40–75)	59.90 ± 7.61 (45–74)
Men,%	15 (65%)	10 (56%)	12 (60%)
Education, y	10 ± 3.15	9.56 ± 3.61	9.30 ± 2.85
Lesionvolume (cc)	6.60 ± 6.23	6.15 ± 6.38	
**Lesion location**			
Left hemisphere	10 (44%)	8 (44%)	
Righthemisphere	13 (56%)	10 (56%)	
FMA-UE	26.26 ± 12.78 (8–42)	28.78 ± 11.95 (7–43)	
MBI	54.04 ± 18.66 (21–81)	57.83 ± 15.96 (21–87)	

*Data are presented as the means ± SD (range) for continuous data and n (%) for categorical data*.

*FMA-UE, Fugl–Meyer Assessment for Upper Extremity; MBI, modified Barthel Index*.

**Table 2:** Comparison of the FMA-UE and the MBI scores between the LF-rTMS and the sham stimulation groups.

	**LF-rTMS**	**Sham rTMS**	***P*_**LF-rTMS vs. sham**_**
**FMA-UE**			
Pre-treatment	26.26 ± 12.78	28.78 ± 11.95	0.524
Post-treatment	49.13 ± 14.36^*^	40.50 ± 10.93^*^^#^	0.041
**MBI**			
Pre-treatment	54.04 ± 18.66	57.83 ± 15.96	0.496
Post-treatment	89.74 ± 11.84^*^	77.17 ± 11.01^*^^#^	0.001

*Data are presented as the means ± SD for continuous data*.

* *Denotes differences within before and after intervention*.

# *Denotes differences between LF-rTMS treatment and sham group at posttreatment*.

*LF-rTMS, low-frequency repetitive transcranial magnetic stimulation; FMA-UE, Fugl–Meyer Assessment for Upper Extremity; MBI, modified Barthel Index*.

### Selection of Resting-State Networks

**Figure 3:**
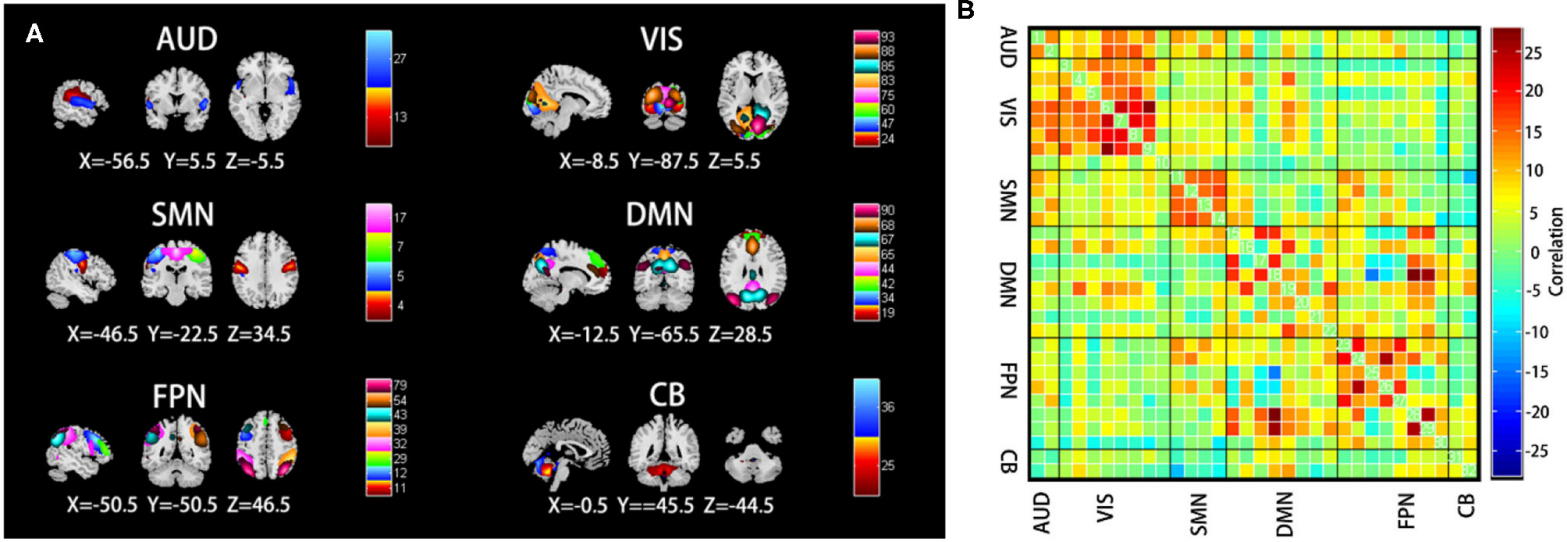
**(A)** Selected the spatial maps of the internal connectivity networks, auditory network (AUN), visual network (VIS), sensorimotor network (SMN), default mode network (DMN), frontoparietal network (FPN), and cerebellar (CB). **(B)** The average resting-state functional connectivity matrix of all the subjects. The color bar represents the *z*-value of the functional connectivity. Hot colors indicate positive correlation, while winter colors indicate negative correlation.

We obtained 32 IC spatial maps through GICA, which formed six physiologically meaningful resting-state brain networks including AUN (ICs 13 and 27); VIS (ICs 24, 47, 60, 75, 83, 85, 88, and 93); SMN (ICs 4, 5, 7, and 17); DMN (ICs 19, 34, 42, 44, 65, 67, 68, and 90); FPN (ICs 11, 12, 29, 32, 39, 43, 54, and 79); and CB (ICs 25 and 36) (Figure 3A). The average FC matrix of all the subjects in the resting state was calculated (Figure 3B).

### Clustering Result

**Figure 4:**
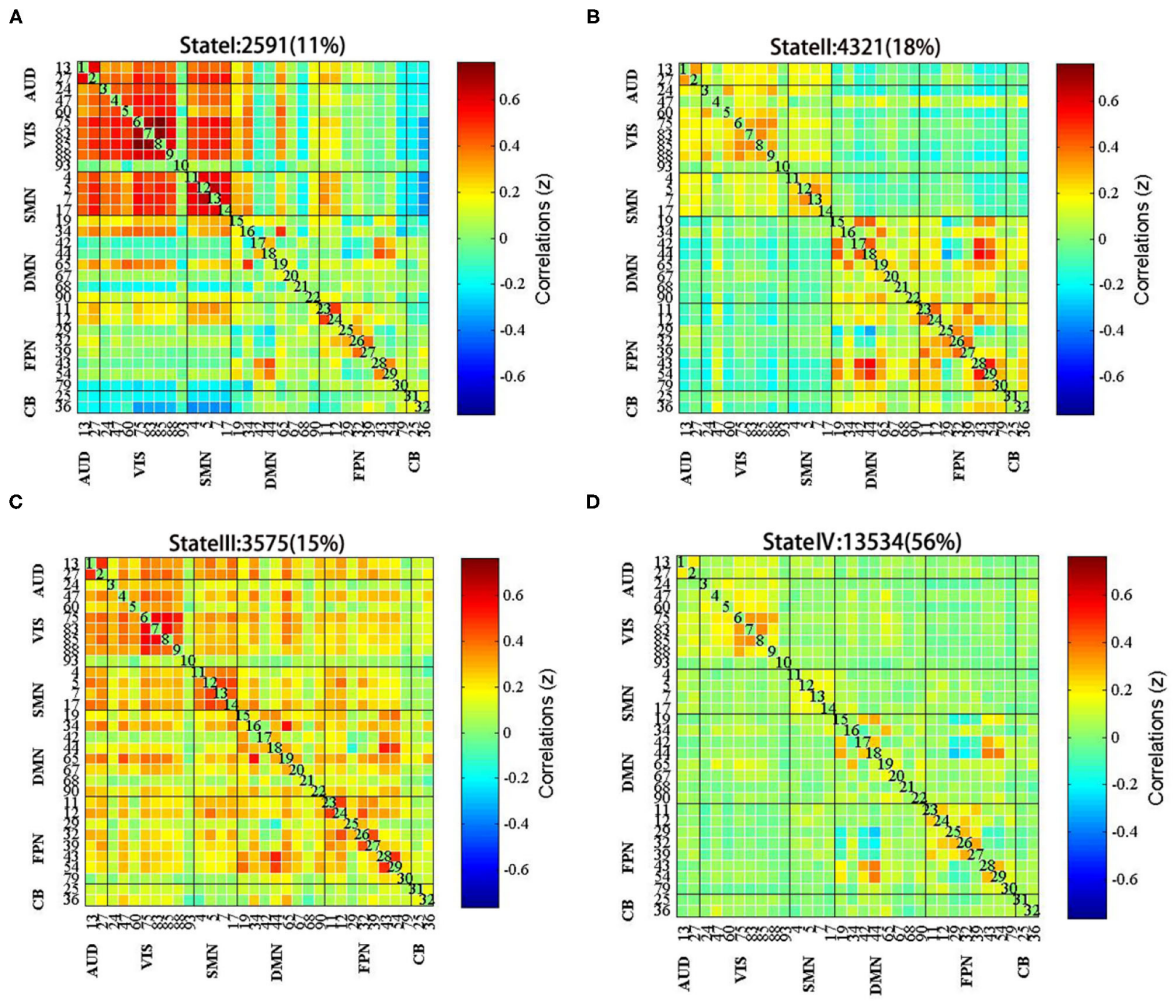
The centroid of each functional network connectivity state with the total occurrences and percentages. **(A)** State I was mainly characterized by strong connections among AUN, VIS, SMN, and DMN; **(B)** State II was mainly characterized by strong connectivity within the perceptual networks and high-order cognitive networks; **(C)** State III was mainly characterized by strong connections between networks; **(D)** State IV was mainly characterized by weak connectivity among VIS, SMN, and FPN.

In this study, the k-means clustering analysis was used to evaluate 153 FC matrixes for each subject and the FC matrixes of all the windows for each subject were used as samples. According to the elbow criterion, four different time-frequency domain matrixes were clustered as follows: state I, which accounted for 11% of all the windows, was mainly characterized by strong connections among AUN, VIS, SMN, and DMN (Figure 4A); state II, which accounted for 18% of all the windows, was mainly characterized by strong connectivity within the perceptual networks (AUN, VIS, SMN) and high-order cognitive networks (DMN, FPN, CB) (Figure 4B); state III, which accounted for 15% of all the windows, was mainly characterized by strong connections between networks (Figure 4C); and state IV, which was the most common, comprising 56% of the study population, was mainly characterized by weak connectivity among VIS, SMN, and FPN (Figure 4D).

### Temporal Characteristics of FC States

**Figure 5:**
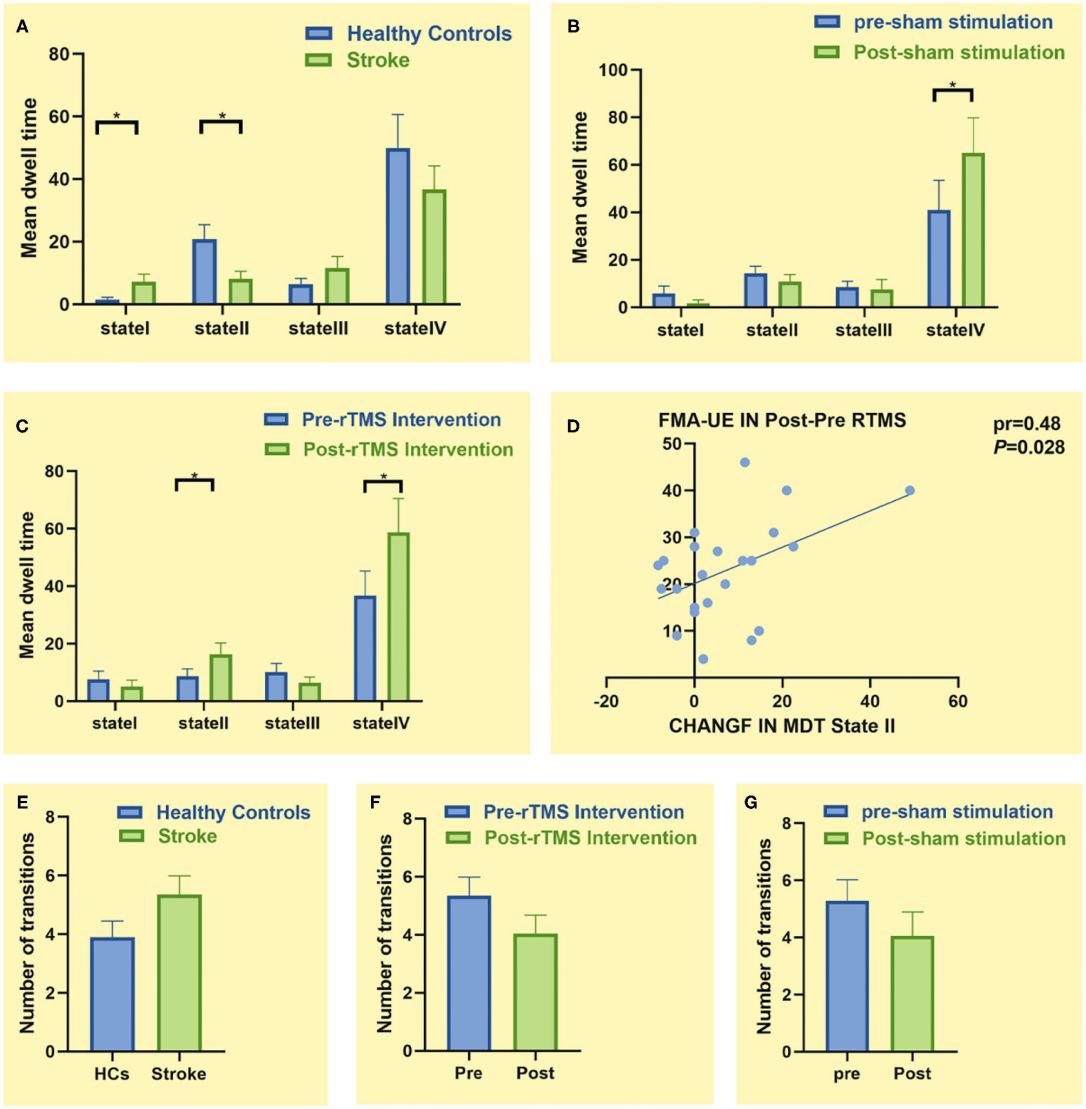
Comparison of temporal characteristics of functional connectivity states between groups. **(A)** Comparison of mean dwell time (MDT) between healthy controls (*n* = 20) and patients with stroke (*n* = 23). **(B)** Comparison of MDT before and after sham stimulation (*n* = 18). **(C)** Comparison of MDT before and after low-frequency repetitive transcranial magnetic stimulation (LF-rTMS) treatment (*n* = 23). **(D)** Correlation between change in the Fugl–Meyer Assessment for Upper Extremity (FMA-UE) score and change in MDT of state II. **(E)** Comparison of number of transitions between healthy controls (*n* = 20) and patients with stroke (*n* = 23). **(F)** Comparison of number of transitions before and after LF-rTMS treatment (*n* = 23). **(G)** Comparison of number of transitions before and after sham stimulation (*n* = 18). * *p* < 0.05

There were differences in MDT between state I and state II groups. In state I, patients with stroke had a higher MDT than HCs (*p* = 0.043, ES = 0.64). Furthermore, the MDT of patients with stroke was shorter than HCs in state II (*p* = 0.015, ES = 0.78), indicating that patients with stroke spend less time separating the sensory-perceptual network and higher-order cognitive control network connections. However, there was no significant difference in MDT between the two groups (HCs vs. patients with stroke) in states III and IV (*p* > 0.05) (Figure 5A).

In state I, the LF-rTMS group (*p* = 0.024, ES = 0.51) and the sham rTMS group (*p* = 0.049, ES = 0.48) spent more time posttreatment than pretreatment (Figures 5B,C). Particularly, patients with stroke spent more time in state II after rather than before LF-rTMS (*p* = 0.015, ES = 0.55) (Figure 5C). This change in MDT was positively correlated with change in the FMA-UE score before and after LF-rTMS treatment (pr = 0.48, *p* = 0.028) (Figure 5D). However, there was no significant difference in MDT before and after treatment in state I and state III (*p* > 0.05).

There were no significant differences in the number of state transitions between patients with stroke and HCs or the stroke subgroups before and after treatment (*p* > 0.05) (Figures 5E–G).

### Spatial Characteristics of FC States

**Figure 6:**
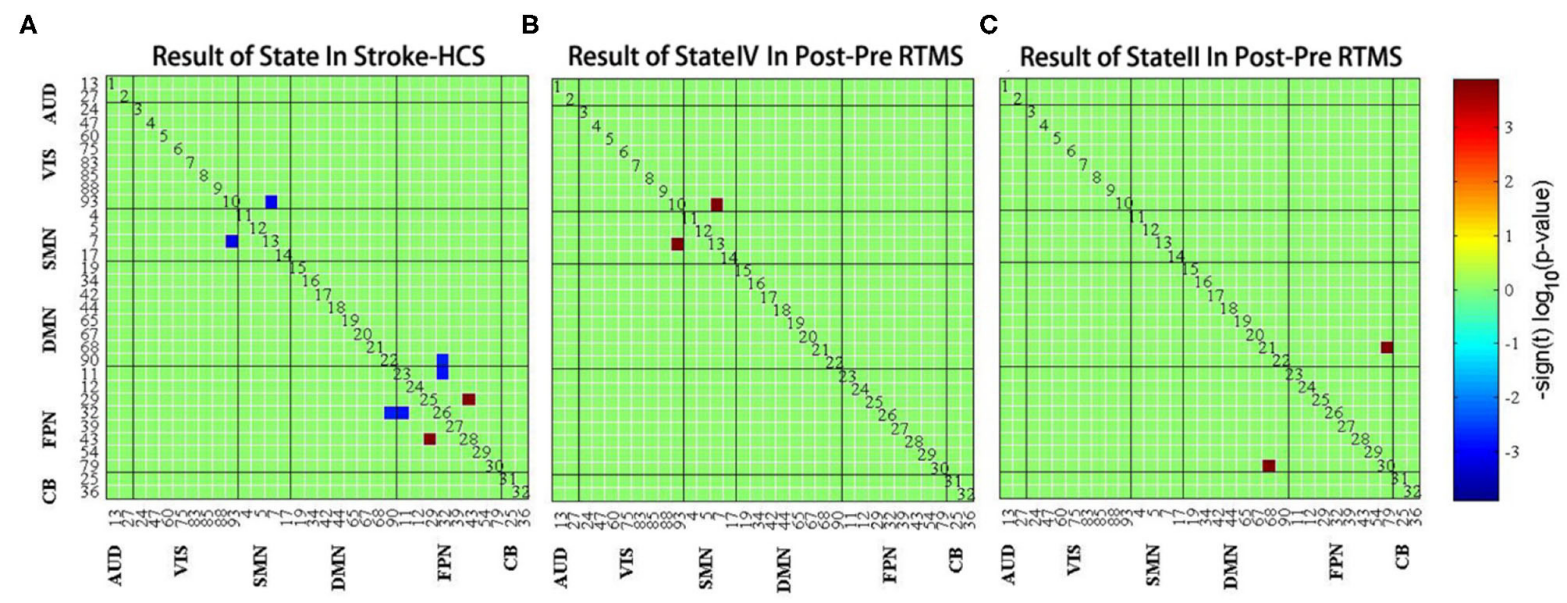
**(A)** The difference of functional connectivity between healthy controls and stroke group in state IV. **(B)** The difference of functional connectivity in state IV before and after LF-rTMS treatment. **(C)** The difference of functional connectivity in state II before and after LF-rTMS treatment. Warm and cool colors indicate increased and decreased connectivity.

Finally, we studied the intragroup and intergroup differences in the connectivity strength of the four connectivity states. Although there were no significant differences between state I and state III, there were significant differences in state II and state IV. Compared to HCs, patients with stroke displayed abnormal decreased FC between SMN and VIS and between FPN and DMN in state IV; subsequently, the enhanced FC was distributed within the FPN [false discovery rate (FDR) corrected, *p* < 0.05] (Figure 6A). Moreover, functional coupling between SMN and VIS and between FPN and DMN increased following LF-rTMS (FDR corrected, *p* < 0.05) (Figures 6B,C).

## Discussion

This study evaluated the characteristics of dynamic brain networks in patients who experienced subcortical stroke and investigated the effect of LF-rTMS combined with rehabilitation training on these networks, focusing on temporal properties (MDT) and on the strength of the FC between networks. In this study, patients with stroke showed an increased MDT in state I and a decreased MDT in state II. Nevertheless, MDT was restored to near HC levels after LF-rTMS combined with rehabilitation. Additionally, the dynamic connectivity of SMN, VIS, DMN, and FPN in patients with stroke was abnormal, yet these changes were partially restored following LF-rTMS combined with rehabilitation. These findings offer new evidence to further understand the neural mechanisms underlying functional recovery after subcortical stroke.

### Imbalance Between Functional Separation and Integration in Patients With Stroke

Focal lesions caused by strokes may not only disrupt connectivity within the motor system, but also change the propensity of the brain in different dynamic connectivity states. We observed that patients with stroke spent a shorter time in state II, which are characterized by high intradomain integration and relatively reduced interdomain connections compared to other states, suggesting reduced functional separation. Previous studies have shown that reduced separation may indicate functional impairment (23). For example, Wang et al. (24) found that patients who experienced a stroke spend significantly less time in a highly separated state compared to HCs, similar to the results of this study. However, Bonkhoff et al. (15) observed that patients with severe motor dysfunction were more likely to transition to a state of intensive regional connectivity, contrary to the results of this study; this difference may be caused by the inclusion of patients at different stages of infarction and varying degrees of injury. The differences in regional time variability among patients with acute, subacute, and chronic stroke need to be further clarified in the future.

Furthermore, this study found that more patients with stroke were in state I than HCs. The main feature of state I was the stronger positive correlation between networks, including AUN, VIS, SMN, and DMN, and this may be a potential compensatory mechanism for internal brain networks. Information integration between large-scale networks after stroke may compensate for the local loss of neural tissue and function (25). Therefore, we believe that this result was due to the inclusion of only moderate and severe patients with stroke. To transform effective cognitive processing into actions, it is necessary to communicate between cognitive control and sensory or motor areas and promote regional processing and reorganization if the motor output is interrupted.

Additionally, we found that the connectivity among SMN, VIS, DMN, and FPN were reduced, while the connectivity was enhanced within the FPN in patients with stroke. SMN contributes to the planning and execution of spontaneous movements (26), while VIS plays a significant role in guiding and adjusting movements. DMN is thought to be involved in the integration of primary perception and higher cognitive processes (27) and FPN is thought to play a regulatory role in the somatosensory cortex and to store related information before the execution of motor behaviors (28). Reduced connectivity between these networks may denote a joint limited neurological function signature following stroke onset, while increased FC within the FPN may be a compensatory mechanism for decreased spontaneous movement or stable motor performance maintenance.

The aforementioned results suggest that there are abnormalities in the separation and integration of brain functions in patients with stroke and confirm the presence of abnormalities in the FC of patients with stroke, providing new insight on neural mechanisms underlying the clinical symptoms of strokes. Although it is unclear what causes dynamic changes in brain states, there is evidence that these changes are related to underlying neuronal activity (29). However, this result cannot be detected in the resting state, demonstrating the advantage of dynamic analysis.

### Repetitive Transcranial Magnetic Stimulation Combined With Rehabilitation Training Promotes Partial Recovery of dFC

As a novel non-invasive neuroregulatory technique, LF-rTMS has been demonstrated to induce changes in neuroplasticity associated with the recovery of motor function. When repeated pulses of LF-TMS of near-threshold intensity are applied to the primary motor cortex, LF-rTMS not only directly regulates transsynaptic activation (I-wave recruitment), but also leads to changes in the excitability of the motor cortex and the preferential recruitment of specific cortical pathways (30, 31). This may change the activity pattern in the relevant regional network that is not conducive to recovery and may, thus, promote synaptic plasticity and recovery of inactivated function. After LF-rTMS combined with rehabilitation training, the MDT of state II patients with stroke became similar to that of HCs and changes in MDT before and after treatment were positively correlated with changes in the FMA-UE scores. This was consistent with our hypothesis that the altered temporal and spatial properties of the FCs were associated with the functional recovery of patients with stroke. This further supports the concept that LF-rTMS combined with rehabilitation training promotes the recovery of the functional connections within the primary sensorimotor domains and the higher cognitive domains. LF-rTMS of the cortical area can influence the activity within the cortico-basal ganglia-thalamo-cortical loop and potentially produce clinical benefits, including improved patient movement features (32), which are consistent with this study. Importantly, our findings may further explain the changes in the frameworks of these regional networks. It is thought that LF-rTMS can induce changes in brain FC that gradually normalize the abnormal network functions with poststroke dyskinesia. This may suggest that dFC method may help to reveal the complex network effects of LF-rTMS during stroke recovery and identify biomarkers of network-level interactions.

Interestingly, a higher dissociation of cognitive control networks was observed after rTMS combined with rehabilitation than before treatment. The MDT of state II in patients with stroke increased significantly after LF-rTMS treatment, which is consistent with the observations of brain modularity (33). Modularity is a significant feature of brain networks and has recently been considered to be a brain plasticity biomarker that is associated with an intervention (34). In healthy aging processes, there is clear evidence that higher modularity is directly proportional to better cognitive function (3). Similarly, this relationship has also been established in the brains of neuropsychiatric patients such as those with traumatic brain injury (35) and schizophrenia (36). Siegel et al. (23) found that increased modularity was associated with better functional recovery after stroke. In this regard, the preference for high-order cognitive control networks in patients with stroke may indicate that LF-rTMS-induced changes are not limited to stimulating motor networks and may also spread to cognitive control networks in an attempt to stimulate multisynaptic effects and facilitate brain plasticity between cognitive areas, thus enabling cognitive enhancement.

Additionally, this study found that the time spent in state IV for both the LF-rTMS and sham rTMS groups at 3 months after infarction was similar to that for HCs. State IV is characterized by a low integration degree and relatively increased connectivity between SMN, VIS, DMN, and FPN. The results of this study indicate that, with the recovery of movement in patients with stroke, new connections between networks were established and the flexibility of networks continuously increased. However, LF-rTMS combined with rehabilitation training was significantly more efficient than sham stimulation, indicating that the former may more easily promote the establishment of new connections between networks and enhance network flexibility, which is better for poststroke functional rehabilitation.

However, this study has several limitations. First, the sample size of this study was small; future multicenter studies with larger study samples and more detailed data to confirm and expand the current findings are warranted. Second, the relationship between dynamic functional connections and structural connections and the former in exercise rehabilitation is unclear; therefore, it is necessary to conduct further research on this matter.

## Summary

In conclusion, this study showed that the temporal properties, including MDT, and spatial properties of functional dynamics are altered in patients who experienced a subcortical stroke. Importantly, these abnormalities are partially improved after LF-rTMS combined with rehabilitation training. Network dynamics may be useful to explore information exchange in the brain and provide complementary evidence to further understand the neural mechanisms underlying the effects of functional recombination and rTMS after subcortical stroke.

## Data Availability Statement

The raw data supporting the conclusions of this article will be made available by the corresponding author.

## Ethics Statement

The studies involving human participants were reviewed and approved by Medical Ethics Committee of the Fuzhou General Hospital (NO. 2015011). The patients/participants provided their written informed consent to participate in this study. Written informed consent was obtained from the individual(s) for the publication of any potentially identifiable images or data included in this article.

## Author Contributions

YQ and XL conceived the idea and drafted the manuscript. XG and ML collected the data. HL and SX analyzed the data. All authors contributed to the final version of the manuscript.

## Funding

This study was supported by the Natural Science Foundation of Fujian (grant number: 2015Y0025) and the Foundation of the 900th Hospital (grant number: 2019Z15).

## Conflict of Interest

The authors declare that the research was conducted in the absence of any commercial or financial relationships that could be construed as a potential conflict of interest.

## Publisher's Note

All claims expressed in this article are solely those of the authors and do not necessarily represent those of their affiliated organizations, or those of the publisher, the editors and the reviewers. Any product that may be evaluated in this article, or claim that may be made by its manufacturer, is not guaranteed or endorsed by the publisher.
