# Corrigendum: Low-frequency repetitive transcranial magnetic stimulation restores dynamic functional connectivity in subcortical stroke

**DOI:** 10.3389/fneur.2023.1272223

**Published:** 2023-08-11

**Authors:** Yin Qin, Xiaoying Liu, Xiaoping Guo, Minhua Liu, Hui Li, Shangwen Xu

**Affiliations:** ^1^Fuzong Clinical Medical College of Fujian Medical University, Fuzhou, Fujian, China; ^2^Department of Rehabilitation Medicine, The 900th Hospital of Joint Logistic Support Force, PLA, Fuzhou, China; ^3^Department of Radiology, The 900th Hospital of Joint Logistic Support Force, PLA, Fuzhou, China

**Keywords:** subcortical stroke, transcranial magnetic stimulation, dynamic functional connectivity, resting-state functional magnetic resonance imaging, time-varying connectivity

In the published article, there was an error in affiliations 1 & 2. These affiliations were mistakenly presented as “^1^Department of Rehabilitation Medicine, The 900th Hospital of Joint Logistic Support Force, PLA, Fuzhou, China. ^2^Department of Rehabilitation Medicine, Fuzong Clinical Medical College of Fujian Medical University, Fuzhou, China.” The correct affiliations were: “^1^Fuzong Clinical Medical College of Fujian Medical University, Fuzhou, Fujian, China. ^2^Department of Rehabilitation Medicine, The 900th Hospital of Joint Logistic Support Force, PLA, Fuzhou, China.”

The authors apologize for this error and state that this does not change the scientific conclusions of the article in any way. The original article has been updated.

